# Effect of Stabilizer States (Solid Vs Liquid) on Properties of Stabilized Natural Rubbers

**DOI:** 10.3390/polym12040741

**Published:** 2020-03-27

**Authors:** Khwanchat Promhuad, Wirasak Smitthipong

**Affiliations:** 1 Specialized center of Rubber and Polymer Materials in agriculture and industry (RPM), Department of Materials Science, Faculty of Science, Kasetsart University, Chatuchak, Bangkok 10900, Thailand; pond_kh@hotmail.com; 2 Office of research integration on target-based natural rubber, National Research Council of Thailand (NRCT), Chatuchak, Bangkok 10900, Thailand

**Keywords:** natural rubber, natural latex, viscosity stabilizer, hydroxylamine sulfate, storage hardening

## Abstract

The main objective of this work is to study the effect of hydroxylamine sulfate or stabilizer states (solid vs liquid) on the storage hardening of natural rubber (NR). Several types of natural rubber samples were prepared: unstabilized NR samples and stabilized NR samples: (i) dry NR with 0.2 and 2.0 parts per hundred rubber (phr) of dry hydroxylamine sulfate, and (ii) natural latex with 0.2 and 2.0 phr of liquid hydroxylamine sulfate. The samples were characterized immediately (time 0) and after 12 weeks of storage at room temperature, respectively. We found that the Mooney viscosity, gel content, and Wallace plasticity of NR without a stabilizer increases with storage hardening for 12 weeks. However, two types of stabilized NR samples represent constant values of those three parameters, because hydroxylamine sulfate inhibits network and gel formation in NR. Interestingly, the mixing states (solid vs liquid) between natural rubber and the stabilizer affect the properties of stabilized NR. This could be explained by the better dispersion and homogeneous nature of liquid stabilizers in natural latex (liquid state), and thus the higher loading of the stabilizer in the liquid state. This is important, as the stabilization of NR properties as a function of time is required by rubber industry. This study is a utilization model from theory to application.

## 1. Introduction

Natural rubber (NR) can be obtained from *Hevea brasiliensis* plant. NR has been widely used across industries for surgical gloves, pillows and mattresses, elastic bands, medical products, and tires, plus many more. NR consists of rubber and non-rubber components, the main component is *cis*-1,4-polyisoprene [1,2,3,4,5]. The non-rubber fraction consists of proteins (ω-terminal) and phospholipids (α-terminal), which are not only attached at each end chain of the polyisoprene, but also included in the serum [6]. When we stored the NR for a prolonged period of time, proteins and phospholipids at the end chain could promote formation of the NR network through protein-protein interactions at the ω-terminal or phospholipid-phospholipid interactions at the α-terminal. The increased of the branch chain or network of NR as a function of time can be determined by the Mooney viscosity via the storage hardening [7]. The storage hardening of NR can be inhibited by a stabilizer. A recent study [8] presented the effect of polar chemicals on the storage hardening of NR. Three types of polar chemicals were used: phenol, diethylene glycol, and hydroxylamine hydrochloride. They found that gel content and Mooney viscosity of NR samples with phenol and diethylene glycol are increased with storage hardening. However, NR samples with hydroxylamine hydrochloride (stabilizer) represent constant values of gel content and Mooney viscosity. Another example study proposed a model wherein the interaction between non-rubber components at the terminal ends of NR molecules and the viscosity stabilizer could be hydrogen bonding [9].

Most of the previous research has presented only the study of dry NR with dry stabilizers, and also fixed the concentration of the stabilizer at only 0.2 parts per hundred rubber (phr) [8,9,10]. It would be interesting to better understand the mixing state between NR and the stabilizer, as well as the concentration of the stabilizer. So, the objective of this present work is to investigate the effect of stabilizer states (dry stabilizer with dry NR and liquid stabilizer with natural latex) at different stabilizer concentrations on the properties of stabilized NR compared to unstabilized NR. This study could allow us to better control the properties of stabilized natural rubber.

## 2. Materials and Methods

### 2.1. Materials

Fresh natural latex was used, with a dry rubber content 29.6 wt.% (Num rubber and latex Co. Ltd, Trang, Thailand), hydroxylamine sulfate (HS) (Sigma-Aldrich, Bangkok, Thailand, preparing by Thai eastern group, Thailand), and toluene (AR grade, RCI Labscan Limited, Bangkok, Thailand).

### 2.2. Preparation of NR Samples

Five types of NR samples (400 g each) were prepared (Table 1): control NR or unstabilized NR, dry NR with 0.2 and 2.0 phr of dry hydroxylamine sulfate (NRD/HS), and natural latex with 0.2 and 2.0 phr of liquid hydroxylamine sulfate (NRL/HS). Then, all the rubber samples were individually masticated by a two-roll mill with a front-rotor speed of 18 rpm and a back-rotor speed of 20 rpm. The mastication for each sample was carried out at 70 °C for 10 min. Finally, the NR samples were characterized after preparation (time 0, or 0 weeks) and also after 12 weeks of storage at room temperature, respectively.

### 2.3. Characterizations

Gel content was measured by dissolving the NR samples in toluene. After that, the solution was kept in the dark at room temperature for one week. Finally, the solution was filtered, weighed, and then calculated with respect to the original weight sample as an equation seen below [1]:

(1) $$\%\mathrm{Gel}\;\mathrm{content}=\frac{\mathrm{Weigth}\;\mathrm{of}\;\mathrm{gel}\times 100}{\mathrm{Weigth}\;\mathrm{of}\;\mathrm{sample}}$$

Mooney viscosity analysis was performed using a Mooney viscometer (viscTECH+, TECHPRO, Columbia City, IN, USA), within a sealed, pressurize, d and heated cavity. A rubber sample was heated at 100 °C for 1 min before analysis. After that, the rubber sample was continuously measured for 4 min by the torque required to keep the rotor rotating at a constant rate as a function of time for reading the Mooney viscosity, which was recorded as torque in newton metre (Nm) [9].

The functional group of rubber samples was determined by attenuated total reflection Fourier transform infrared (ATR-FTIR VERTEX 70, Bruker, Billerica, MA, USA). Rubber samples were cut into small pieces and put on a Ge crystal probe at 600–4000 cm^−1^ [9].

The Wallace plasticity value was determined according to the International Organization for Standardization (ISO 2007). The small rubber disk was compressed between two platens at 100 °C for 15 s at a fixed thickness of 1 mm. After this preheating period, the rubber specimen was subjected to a constant compressive force of 100 N for 15 additional seconds. The thickness of the specimen at the end of this period was taken as the Wallace plasticity value. Plasticity retention index (PRI) is a measure of the resistance of raw natural rubber to oxidation. The oxidation effect was assessed by measuring the plasticity before aging (Po) and after aging for 30 min in the MonTech aging oven for plasticity testing at 140 °C (P_30_) [11]:

(2) $$\mathrm{PRI}=\frac{P_{30}\times 100}{\mathrm{Po}}$$

The processability under strain sweep modes of the rubber samples was investigated by a rubber processing analyzer (RPA 2000, Alpha Technologies, Hudson, OH, USA). The strain sweep mode was in the range of 0.5–100%, the process was carried out at 100 °C and 1 Hz [9].

The viscoelastic properties of uncrosslinked rubbers were determined by dynamic mechanical analysis (DMA1, Mettler Toledo, Columbus, OH, USA) based on Williams–Landel–Ferry (WLF) analysis. The time-temperature superposition principle was used to establish a master curve of the storage modulus (E’) as a function of reduced frequency at a reference temperature *T*_ref_ of 298 K. Shift factors (*a_T_*) for establishment of master curves were determined according to the universal WLF equation, where *c_1_* and *c_2_* are constants depending on the nature of the elastomer and the reference temperature [12]:

(3) $$\log a_{T}=\frac{\left(-c_{1}(T_{\exp}-T_{\mathrm{ref}}\right)\;}{\left(c_{2}+(T_{\exp}-T_{\mathrm{ref}}\right)}$$

## 3. Results and Discussion

**Figure 1:**
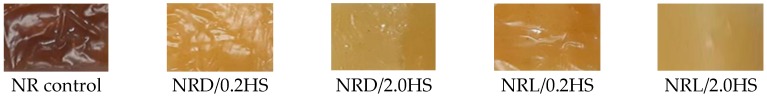
The natural rubber samples used in this study for 0 weeks: NRD/0.2HS means dry NR with dry hydroxylamine sulfate 0.2 phr, NRD/2.0HS means dry NR with dry hydroxylamine sulfate 2.0 phr, NRL/0.2HS means natural latex with liquid hydroxylamine sulfate 0.2 phr, NRL/2.0HS means natural latex with liquid hydroxylamine sulfate 2.0 phr.

Concerning to the visual aspect of samples (Figure 1), we found that the control NR without any stabilizer had a dark brown color at 0 weeks. However, the dry rubber with dry HS samples had a light brown color. Moreover, the latex with liquid 2.0 phr of the HS sample seemed to be the lightest brown color. After 12 weeks (data not shown), the color of the control NR became darker. However, the color of all the rubbers with stabilizers were stable.

**Figure 2:**
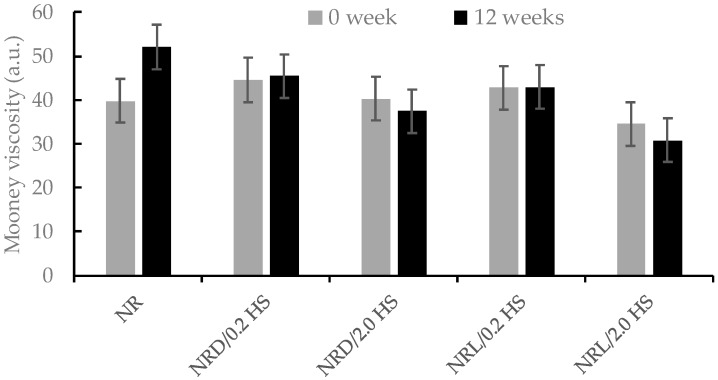
The Mooney viscosity of the samples at 0 and 12 weeks.

We investigated the Mooney viscosity of the samples, which depends on the macrostructure of the rubber samples. The results of Mooney viscosity of the samples at various storage times are shown in Figure 2. At time 0, hydroxylamine sulfate additive stabilized both dry rubber and latex which, are NRD/0.2HS, NRD/2.0HS, NRL/0.2HS, and NRL/2.0HS, respectively. When the amount of hydroxylamine sulfate in rubber was increased, the Mooney viscosity of sample was decreased. However, the latex samples showed a Mooney viscosity lower than the samples from dry rubber. After 12 weeks, the Mooney viscosity increased for the control sample, which is a sign of the storage hardening phenomenon of natural rubber [13]. However, the NR samples supplemented with hydroxylamine sulfate were stable (NRD/0.2HS, NRD/2.0HS, NRL/0.2HS, and NRL/2.0HS). Considering the effect of the viscosity stabilizer, all NR samples with the viscosity stabilizer HS (0 and 12 weeks) had a more stable Mooney viscosity than those without. Interestingly, when the liquid hydroxylamine sulfate was mixed with natural latex (NRL/HS), its Mooney viscosity seemed to be lower than that of the NRD/HS sample at a given concentration. This could be explained by the better dispersion and homogeneous nature of liquid hydroxylamine sulfate in natural latex (liquid state) compared to the mixing of HS and NR in a dry state.

**Figure 3:**
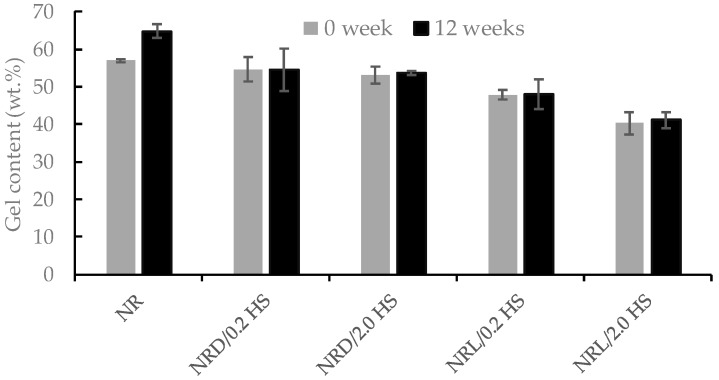
Gel content of NR samples for times 0 and 12 weeks.

The gel content of the rubber samples was also interesting for investigating the effect of stabilizer states (solid vs liquid), and the amounts of gel content are shown in Figure 3. At time 0, the control NR sample has a gel content slightly higher than the samples with hydroxylamine sulfate (NRD/0.2HS, NRD/2.0HS, NRL/0.2HS, and NRL/2.0HS). After 12 weeks, the NR control had a higher gel content, resulting from gel formation during prolonged storage [1,14,15]. The gel content depends on proteins and lipids that are the major components in the formation of the gel fraction during NR storage [16]. In contrast, the gel content was stable for NRD/0.2HS, NRD/2.0HS, NRL/0.2HS, and NRL/2.0HS. The effect of proteins and lipids causes a gel network that is called storage hardening in NR. However, the viscosity stabilizer hydroxylamine sulfate inhibited gel formation in the NR samples. Again, the NRL/HS sample had a lower gel content than the NRD/HS sample at a given concentration. So, the effects of stabilizers in liquid state were more pronounced. This result is in good agreement with the results of the Mooney viscosity analysis.

**Figure 4:**
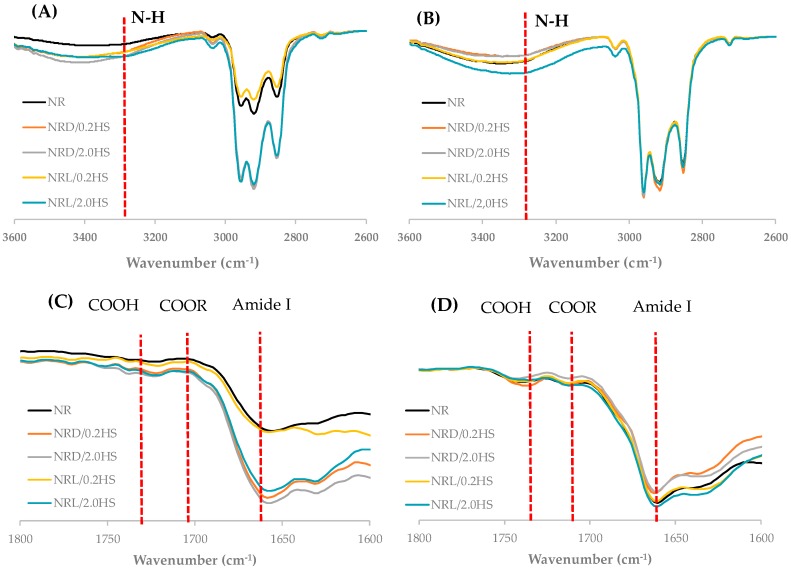
FTIR spectra of NR with hydroxylamine sulfate samples: (**A**) and (**C**) at 0 weeks, (**B**) and (**D**) at 12 weeks.

The chemical compositions of the NR samples and non-rubber components were analyzed by attenuated total reflection Fourier transform infrared or ATR-FTIR (Figure 4). The FTIR spectra of the NR with hydroxylamine sulfate samples presented at 3600–2600 cm^−1^ and 1800–1600 cm^−1^. At time 0, samples showed peaks for the amine group (N–H) at 3280 cm^−1^, the fatty acid ester group at 1740 cm^−1^, the aldehyde group at 1710 cm^−1^, and the amide I group at 1660–1630 cm^−1^ [13]. After 12 weeks, all the peaks of the control NR sample were increased, and this result is in good agreement with a previous study [9]. For the stabilized NR samples, there was almost no change in the FTIR spectra, except NRL/0.2HS, which was close to the control NR sample. However, the ATR-FTIR technique focuses on a few microns from the sample surface, which is quite different from the bulk results of the Mooney viscosity and gel content analyses between control NR and NRL/0.2HS, in particular with this low concentration of liquid stabilizer (NRL/0.2HS), which probably would dilute the ATR-FTIR measurement.

We were also interested in the elasticity of rubber samples, which could be determined by the original Wallace plasticity (Po). When the Po value of rubber is high, its elasticity is high [8]. The Po value of control NR sample increased after being stored for 12 weeks, whereas the other NR samples with hydroxylamine sulfate had a constant Po value (Table 2). However, the NRL/HS samples possessed lower Po values than NRD/HS, no matter the concentration of HS and the storage time. This result is in good agreement with the results of the Mooney viscosity and gel content analyses. The proteins and phospholipids in NR represent the network and the gel formation, which causes the increase in Po. This phenomenon is called storage hardening [11]. Unlike the NR with stabilizer samples [16], they represented almost stable Po values as a function of time within the uncertainty values (± 5 a.u.).

The results of Plasticity Retention Index (PRI) are also presented in Table 2. This test estimates the resistance to oxidation and breakage of rubber molecules at higher temperatures. Similar to the Po results, the PRI values of NR with stabilizer samples were stable, unlike the PRI value of the control NR sample, which decreased after 12 weeks of storage time [13].

**Figure 5:**
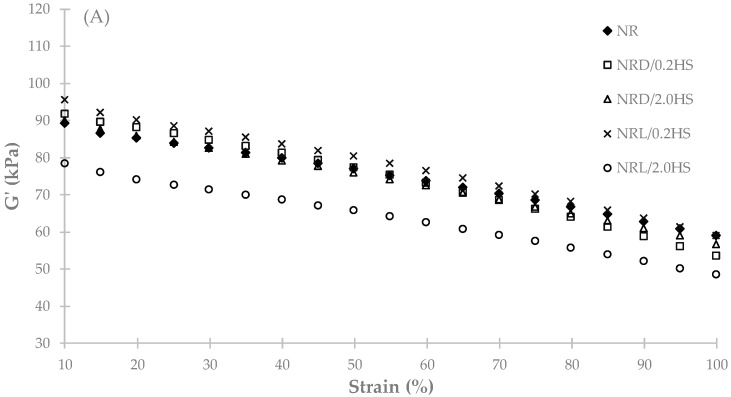

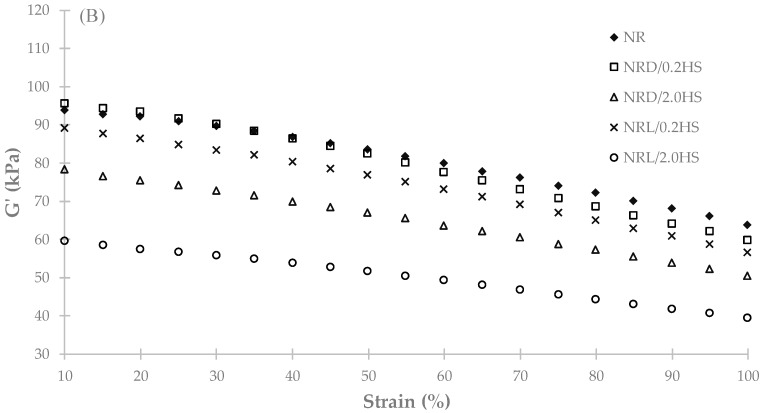
Relationship between storage modulus (G’) and the strain of NR with hydroxylamine sulfate samples for 0 weeks (**A**) and 12 weeks (**B**).

Concerning the rigidity or storage modulus (G’) of NR samples as a function of strain, its values for 0 and 12 weeks can be seen in Figure 5. At time 0, the results show that all the NR samples with or without stabilizer possessed almost the same level of rigidity (G’), whereas NRL/2.0HS had the lowest G’. This result is in good agreement with the result of the Mooney viscosity analysis. After 12 weeks, the rigidity of the control NR increased slightly compared to that at 0 weeks. However, the rigidity of NR with HS almost decreased compared to that of 0 weeks. This may be explained by the increased Mooney viscosity and gel content of the control NR sample after 12 weeks.

**Figure 6:**
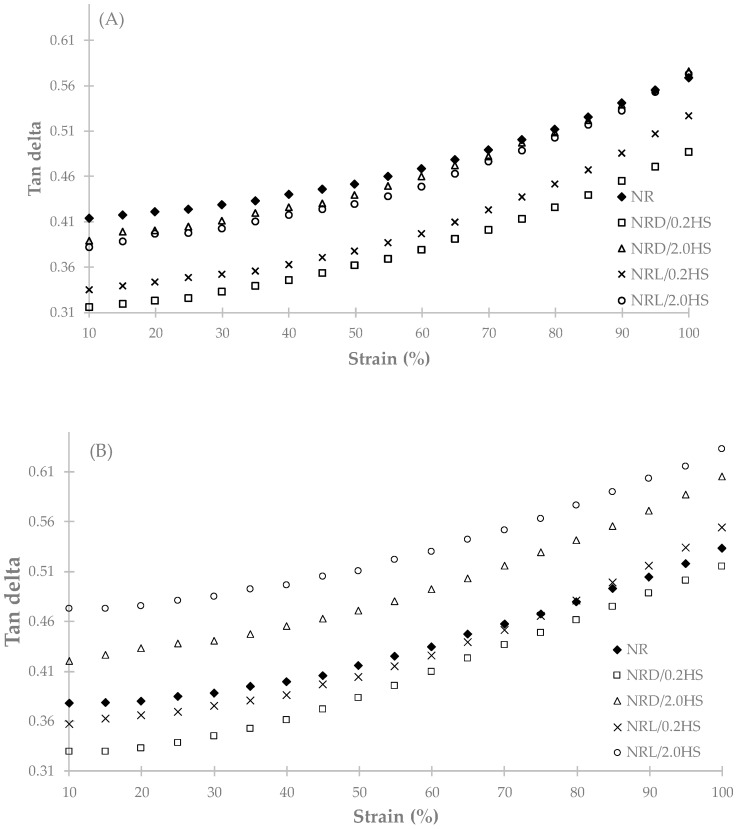
Relationship between tan delta and the strain of NR with hydroxylamine sulfate samples for 0 weeks (**A**) and 12 weeks (**B**).

The tan delta is the ratio of the loss modulus (G”) and storage modulus (G’), a high tan delta value means the rubber samples have lost more energy or have a higher heat build-up. At 0 weeks, the control NR sample had a tan delta level close to those of NR with higher amounts of stabilizer (2 phr), whereas the tan delta of NR with lower amounts of stabilizer (0.2 phr) was lower (Figure 6). At 12 weeks, all samples with hydroxylamine sulfate had an increasing tan delta. However, the control NR sample had the decreasing of tan delta because of the increasing network structure and longer molecular chains, which reduced the molecular friction or heat build-up [9].

**Figure 7:**
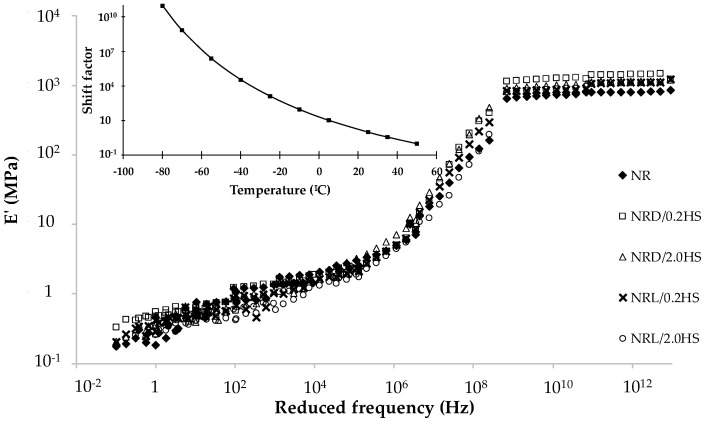
Storage modulus (E’) versus reduced frequency master curves of NR with or without hydroxylamine sulfate for 12 weeks, the insert figure is the shift factor as a function of temperature for control NR sample.

One can study the viscoelastic properties of NR samples as master curves (time-temperature superposition) determined by dynamic mechanical analysis, which represents the storage modulus of rubber samples as a function of reduced frequency. The mean values of the constants *c_1_* and *c_2_* for all NR samples (8.50 and 186.50) were rather in good agreement with the Ferry (5.94 and 151.60) reference [12]. The shift factor (*a_T_*) as a function of temperature for the control NR sample is presented as an insert in Figure 7, and the other samples apply the same shift factor. We found that all the NR samples with or without the stabilizer [16] possessed similar viscoelastic properties (Figure 7) at 12 weeks, which was also similar to the results for 0 weeks.

Figure 7 presents the master curves in three zones: glassy plateau at high reduced frequency, transition state, and rubbery plateau at low reduced frequency. Rubber molecules are frozen in the glassy state below the glass transition temperature. The rotation around the molecular bonds increases with increasing the temperature to reach a rubbery state, which means that the rubber molecules become entangled.

## 4. Conclusions

A molecular chain of NR is mostly *cis*-1,4-polyisoprene, whereas the terminal chains are divided into proteins and phospholipids (non-rubber components), which causes storage hardening of NR as a function of time. We investigated the effects of stabilizer states (solid vs liquid), concentration of stabilizers (0.2 and 2.0 phr), and storage time (0 and 12 weeks) on the properties of stabilized NR compared to unstabilized NR. We found that NR with hydroxylamine sulfate (0.2 and 2.0 phr) had more constant values of gel content, Mooney viscosity, and Wallace plasticity compared to those of unstabilized NR. The result of processability under strain sweep is in good agreement with the result of the Mooney viscosity analysis. Interestingly, the mixing condition between NR and the stabilizer in solid vs liquid states affects the properties of stabilized NR. When we mixed NR with stabilizer in a liquid state, this type of sample obtained better performance to keep a lower and more stable Mooney viscosity compared to the sample mixed in a dry state. This opens up more applications of stabilized natural rubber in industry, in particular, it shows a better compromise between the processing and properties.

## Figures and Tables

**Table 1:** List of natural rubber (NR) samples used in this study (phr means parts per hundred rubber).

Name	Samples
Control NR	Unstabilized NR
NRD/0.2 HS	Dry NR with dry hydroxylamine sulfate 0.2 phr
NRD/2.0 HS	Dry NR with dry hydroxylamine sulfate 2.0 phr
NRL/0.2 HS	Natural latex with liquid hydroxylamine sulfate 0.2 phr
NRL/2.0 HS	Natural latex with liquid hydroxylamine sulfate 2.0 phr

**Table 2:** The original Wallace plasticity (Po) and Plasticity Retention Index (PRI) of the rubber samples at times 0 and 12 weeks.

Sample Name	Po (± 5 a.u.)		PRI (± 5 a.u.)	
	0 Weeks	12 Weeks	0 Weeks	12 Weeks
NR	20.0	28.0	85.0	67.9
NRD/0.2 HS	28.5	32.0	61.4	59.4
NRD/2.0 HS	22.0	21.5	54.6	51.2
NRL/0.2 HS	22.5	24.5	62.2	59.2
NRL/2.0 HS	18.5	18.0	56.8	54.2
